# Immunological status of the olfactory bulb in a murine model of Toll-like receptor 3-mediated upper respiratory tract inflammation

**DOI:** 10.1186/s12974-022-02378-1

**Published:** 2022-01-10

**Authors:** Ryoji Kagoya, Makiko Toma-Hirano, Junya Yamagishi, Naoyuki Matsumoto, Kenji Kondo, Ken Ito

**Affiliations:** 1 Department of Otolaryngology, Faculty of Medicine, Teikyo University, 2-11-1, Kaga, Itabashi-ku, Tokyo, 173-8605 Japan; 2 Department of Otorhinolaryngology-Head and Neck Surgery, Graduate School of Medicine, The University of Tokyo, 7-3-1, Hongo, Bunkyo-ku, Tokyo, 113-0033 Japan; 3 Department of Otolaryngology and Head and Neck Surgery, Kameda Medical Center, 929, Higashi-cho, Kamogawa, Chiba 296-8602 Japan

**Keywords:** Poly(I:C), TLR3, Olfactory bulb, Microglia, Proinflammatory cytokines

## Abstract

**Background:**

Postviral olfactory dysfunction (PVOD) following a viral upper respiratory tract infection (URI) is one of the most common causes of olfactory disorders, often lasting for over a year. To date, the molecular pathology of PVOD has not been elucidated.

**Methods:**

A murine model of Toll-like receptor 3 (TLR3)-mediated upper respiratory tract inflammation was used to investigate the impact of URIs on the olfactory system. Inflammation was induced via the intranasal administration of polyinosinic–polycytidylic acid (poly(I:C), a TLR3 ligand) to the right nostril for 3 days. Peripheral olfactory sensory neurons (OSNs), immune cells in the olfactory mucosa, and glial cells in the olfactory bulb (OB) were analyzed histologically. Proinflammatory cytokines in the nasal tissue and OB were evaluated using the quantitative real-time polymerase chain reaction (qPCR) and enzyme-linked immunosorbent assay (ELISA).

**Results:**

In the treated mice, OSNs were markedly reduced in the olfactory mucosa, and T cell and neutrophil infiltration therein was observed 1 day after the end of poly(I:C) administration. Moreover, there was a considerable increase in microglial cells and slight increase in activated astrocytes in the OB. In addition, qPCR and ELISA revealed the elevated expression of interleukin-1 beta, interleukin-6, tumor necrosis factor-alpha, and interferon-gamma both in the OB and nasal tissue.

**Conclusions:**

Taken together, the decreased peripheral OSNs, OB microgliosis, and elevated proinflammatory cytokines suggest that immunological changes in the OB may be involved in the pathogenesis of PVOD.

## Background

The nasal mucosa acts as a frontline interface between the environment and the nasal wall, where virally infected nasal epithelial cells trigger various immune responses, such as the secretion of chemokines and cytokines and the accumulation of immune cells [1]. Viral infections often impact the respiratory system in various ways, such as exacerbating asthma [2] and eliciting rhinosinusitis [3] and olfactory dysfunction [4]. Postviral olfactory dysfunction (PVOD), which is defined as any olfactory disorder that occurs following a viral upper respiratory tract infection (URI), is one of the most common causes of damage to the olfactory system [5].

Although the mechanisms behind the molecular pathology of PVOD have not yet been fully elucidated, there are some reports about the pathophysiology of the disease. For example, Jafek et al. [6] confirmed through a histological analysis that the olfactory epithelium (OE) of patients with PVOD was markedly disorganized. Yamagishi et al. [7] revealed that the numbers of olfactory sensory neurons (OSNs) and nerve bundles in the olfactory mucosa of patients with PVOD were decreased. They also reported a correlation between the density of OSNs and the prognosis of olfactory dysfunction [7]. Using positron emission tomography, Kim et al. [8] discovered significant hypometabolism in the right piriform cortex, bilateral amygdala, and parahippocampal areas of patients afflicted with the disease. Furthermore, Kanaya et al. [9] demonstrated a decrease in the density of mature OSNs in a murine model of polyinosinic–polycytidylic acid (poly(I:C))-induced URI and revealed that the damage to the OSNs by poly(I:C) was attributed mainly to the cytotoxic effect of elastase released by neutrophils infiltrating the olfactory mucosa.

Poly(I:C), a synthetic analog of viral double-stranded RNA, stimulates Toll-like receptor 3 (TLR3) and thereby induces the immune response associated with viral infections, such as the loss of epithelial integrity and production of inflammatory cytokines [10–12]. TLRs, which are a family of pattern recognition receptors, are activated upon their recognition of pathogen-associated molecular patterns and mediate the initiation of rapid innate immune responses [11, 12]. TLR3, a subtype of the TLR family, is expressed on respiratory epithelial cells, OSNs, and antigen-presenting cells, such as dendritic cells and macrophages [9, 10].

Although the immune responses of the respiratory epithelium or peripheral OSNs to TLR3 stimulation have been reported [9, 13], there are as yet no published studies on the impact of TLR3-mediated upper respiratory tract inflammation on the olfactory bulb (OB). Despite that the turnover cycle of OSNs is reported to be 1 month [14], olfactory disorders associated with URIs often take over a few years to improve [15, 16]. These facts suggest that it is possible that there are some changes in the OB of patients with PVOD. Other researchers have demonstrated increases in both the density of microglial cells and the expression of proinflammatory cytokines in the OB of Alzheimer’s or Parkinson’s disease or neurodegenerative diseases with olfactory disorders [17, 18].

In the present study, we concentrated on the status of the glial cells and proinflammatory cytokines in the OB of mice with poly(I:C)-induced upper respiratory tract inflammation.

## Methods

### Mice

C57BL/6 male mice were purchased from CLEA Japan (Tokyo, Japan). All the mice were 7 weeks of age at the start of the experiment. They were housed in a temperature-controlled facility, where they were maintained on a 12 h light/dark cycle and given free access to diet and water. All procedures were approved by the Teikyo University Animal Care Committee and conducted in accordance with the Teikyo University guidelines for the handling and care of laboratory animals.

### Experimental protocol

**Fig. 1:**
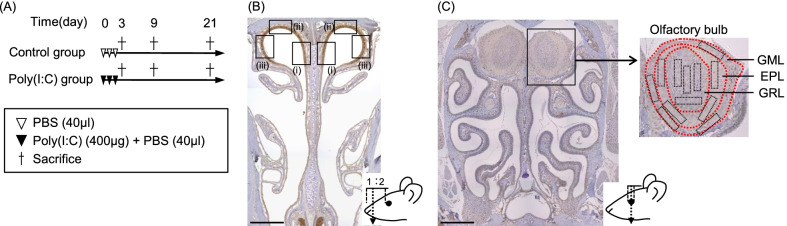
**A** Protocol for generating the murine model of TLR3-mediated upper respiratory tract inflammation and images for analysis. Two groups of mice were received either poly(I:C), or vehicle to the right nostril. **B** Images and levels of cutting positions for the evaluation of OSNs and immune cells. The areas for evaluation of OSNs and immune cells are the nasal septum (i), the tegmen of the nasal cavity (ii), and the lateral wall of the nasal cavity (iii). Scale bar = 500 μm. **C** Images and levels of cutting positions for the evaluation of OB microglia/macrophages and astrocytes. The areas for the evaluation of the OB are GRL, EPL, and GML (Scale bar = 500 μm). *EPL* external plexiform layer, *GML* glomerular layer, *GRL* granular layer, *OB* olfactory bulb, *OSNs* olfactory sensory neurons, *poly(I:C)* polyinosinic–polycytidylic acid, *TLR* Toll-like receptor

The mice were divided into two groups, namely, Control and Poly(I:C). The study protocol is depicted in Fig. 1A. In brief, mice in the Poly(I:C) group received 400 µg of poly(I:C) (Sigma-Aldrich, Missouri, USA) in 40 μL of phosphate-buffered saline (PBS), administered to the right nostril each day for 3 days. The animals in the Control group were administered 40 μL of PBS to the right nostril each day for 3 days. All the mice were sacrificed at day 3, day 9, or day 21. The body weights of all mice were monitored daily. In each experiment, five mice per group were used.

### Buried food test

For evaluating the olfactory function, buried food tests were performed according to Yang et al.’s method, with some modifications [19]. Briefly, a clean cage was prepared (44 cm *L* × 29 cm *W* × 20 cm *H*) containing a 1 cm depth of clean bedding. The subject mouse was transferred to the test cage, allowed to acclimatize for 5 min, and then returned to the original cage. After the mouse had been returned to the home cage, one piece of cookie (Tabekko Dobutsu Biscuits, Ginbisu, Tokyo, Japan) was buried in a random corner, 1 cm beneath the surface of the test cage. The subject mouse was then transferred to the center of the test cage and latency was measured between the timepoint of transfer and the subject mouse finding the buried food.

### Fixation and tissue preparation

The mice were deeply anesthetized using a combination of ketamine and xylazine, following which they were fixed by cardiac perfusion with 10% neutral-buffered formalin, and then decapitated. After the lower jaws had been discarded, the remaining parts were further fixed in 10% neutral-buffered formalin for 1 week at ambient temperature (*T*_A_). Thereafter, the specimens were decalcified in Kalkitox (Wako, Osaka, Japan) for 4 h at *T*_A_, following which they were washed, dehydrated in a graded ethanol series, and embedded in paraffin. Serial coronal sections (4 μm thick) were cut at two levels: (1) at the anterior third of the naso-ocular distance (i.e., one third of the distance from the nose tip to the anterior border of the eyes) (Fig. 1B); and (2) at the center of the eyes (Fig. 1C). The prepared sections were mounted on MAS-coated slides (Matsunami Glass, Osaka, Japan) and subsequently subjected to hematoxylin and eosin staining or immunostaining as described below.

### Immunohistochemistry

To identify matured OSNs, T cells, neutrophils, microglia/macrophages, microglia, and activated astrocytes in the specimens, the following primary antibodies were used, respectively: anti-olfactory marker protein (OMP; goat polyclonal, Wako, Osaka, Japan), anti-cluster of differentiation 3 (CD3; rabbit monoclonal, clone SP7; Nichirei, Tokyo, Japan), anti-lymphocyte antigen 6 complex locus protein G6D/lymphocyte antigen 6 complex locus protein G6C (Ly-6G/Ly-6C; rat monoclonal, clone NIMP-R14; abcam, Cambridge, England), anti-ionized calcium-binding adaptor molecule 1 (Iba-1; goat polyclonal, Wako, Osaka, Japan), anti-transmembrane protein 119 (TMEM119; rabbit-polyclonal, Gene Tex,, California, USA), and anti-glial fibrillary acidic protein (GFAP; rat monoclonal, clone 2.2B10; Invitrogen, Massachusetts, USA) were used. Rabbit IgG isotype-matched control antibody (Invitrogen, Massachusetts, USA), rat IgG isotype-matched control antibody (Invitrogen, Massachusetts, USA), and goat IgG isotype-matched control antibody (Invitrogen, Massachusetts, USA) were used as negative controls.

After deparaffinization and rehydration of the tissue sections, they were subjected to heat-induced antigen retrieval. For OMP and CD3 immunostaining, a citrate buffer solution (pH 6.0; S2369, Agilent Technologies, California, USA) was used. For Ly-6G/Ly-6C and TMEM119 immunostaining, a modified citrate buffer solution (pH 6.1; S1699, Agilent Technologies, California, USA) was used. For Iba-1 and GFAP immunostaining, a target retrieval solution (pH 9.0; S2368, Agilent Technologies, California, USA) was used. Endogenous peroxidase activity was blocked by treatment with 3% hydrogen peroxidase (nacalai tesque, Kyoto, Japan) in methanol for 10 min at *T*_A_. Next, the sections were first incubated in a serum-free protein block (Agilent Technologies, California, USA) at *T*_A_ for 30 min to block nonspecific antibody binding and then incubated with the respective primary antibodies described above at 4 °C overnight. Then, after several washes in PBS, the sections were incubated with horseradish peroxidase-conjugated anti-rat, anti-rabbit, or anti-goat IgG antibodies (Simplestain MAX-PO [Rat], [R], or [G] Nichirei, Tokyo, Japan) at *T*_A_ for 30 min. Following three more washes in PBS, color development was achieved with diaminobenzidine (Simplestain DAB, Nichirei, Tokyo, Japan). Then after washing with distilled water, the sections were counterstained with hematoxylin, dehydrated, and mounted. Histological images of the specimens were obtained by fluorescence microscopy (BZ9000, Keyence, Osaka, Japan).

### Immunofluorescence

To evaluate the ratio of TMEM119^+^ cells (microglia) to Iba-1^+^ cells (microglia and macrophages), immunofluorescence staining was performed. After deparaffinization and rehydration, heat-induced antigen retrieval using a target retrieval solution (pH 6.1; S1699, Agilent Technologies, California, USA) was performed. Next, the sections were incubated in a serum-free protein block (Agilent Technologies, California, USA) at *T*_A_ for 30 min and then incubated with anti-Iba-1 antibody and anti-TMEM119 antibody at 4 °C overnight. Primary-stained sections were incubated with appropriate Alexa Fluor-conjugated secondary antibodies for 30 min at *T*_A_. The following secondary antibodies were used, respectively: Donkey anti-goat IgG secondary antibody Alexa Fluor 488 (Invitrogen, Massachusetts, USA) and Donkey anti-rabbit IgG secondary antibody Alexa Fluor 568 (Invitrogen, Massachusetts, USA). After washing with PBS, sections were mounted in Vectashield mounting medium with DAPI (Vector Laboratories, California, USA). Images of specimens were obtained by fluorescence microscopy (BZ9000, Keyence, Osaka, Japan).

### Histological analysis

To analyze changes in the OE, coronal tissue sections at the level of the anterior third of the naso-ocular distance were used. The number of OMP-positive cells per 100 μm length and the thickness were determined in three areas of the OE: nasal septum, tegmen, and lateral part (shown in Fig. 1B).

To determine the degree of immune cell infiltration into the olfactory nasal mucosa, the number of infiltrated cells was counted manually on the sections cut at the level of the anterior third of the naso-ocular distance. The number of each type of immune cell per 200 μm length of the OE was counted in the following three areas: nasal septum, tegmen, and lateral part (Fig. 1B). Then, the mean cell count for each immune cell was calculated.

To evaluate the glial cells of the OB, the numbers of microglia/macrophages (Iba-1^+^ cells), microglial cells (TMEM^+^ cells), and activated astrocytes (GFAP^+^ cells) were counted manually on the sections cut at the level of the center of the eyes. Each type of glial cell was counted in the area of rectangle combination, and the total value was calculated as the cell count per 0.2 mm^2^. In this way, the number of glial cells per 0.2 mm^2^ of the OB was counted in the following three areas: the granule layer (GRL), external plexiform layer (EPL), and glomerular layer (GML) (shown in Fig. 1C).

### RNA extraction and real-time reverse transcriptase PCR analysis

For RNA extraction, the nasal mucosa was resected microscopically, minced, and lysed in TRIzol reagent (Invitrogen, Massachusetts, USA). Then, total RNA was extracted according to the manufacturer’s instructions and reverse-transcribed using the PrimeScript RT Master Mix (Takara Biotechnology, Shiga, Japan). Then, quantitative real-time polymerase chain reaction (qPCR) was carried out on an Applied Biosystems 7500 Fast Real-Time PCR system. The following primers and probes for the interleukin-1 beta (*Il1b*), interleukin-6 (*Il6*), tumor necrosis factor (*Tnf*), interferon gamma (*Ifng*), TLR3 (*Tlr3*), and glyceraldehyde 3-phosphate dehydrogenase (*Gapdh*) genes were used:

*Il1b*: sense 5ʹ-tccaggatgaggacatgagcac-3ʹ, antisense 5ʹ-gaacgtcacacaccagcaggtta-3ʹ.

*Il6*: sense 5ʹ-ccacttcacaagtcggaggctta-3ʹ, antisense 5ʹ-tgcaagtgcatcatcgttgttc-3ʹ.

*Tnf*: sense 5ʹ-actccaggcggtgcctatgt-3ʹ, antisense 5ʹ-gtgagggtctgggccatagaa-3ʹ.

*Ifng*: sense 5ʹ-cggcacagtcattgaaagccta-3ʹ, antisense 5ʹ-gttgctgatggcctgattgtc-3ʹ.

*Tlr3*: sense 5ʹggtacatcacgcagttcagcaag-3ʹ, antisense 5ʹ-ggccagttcaagatgcaatgag-3ʹ.

*Gapdh*: sense 5ʹ-tgtgtccgtcgtggatctga-3ʹ, antisense 5ʹ-ttgctgttgaagtcgcaggag-3ʹ.

The gene expression levels were calculated as the difference between the cycle threshold (Ct) value of the target gene and that of *Gapdh* (ΔCt) using the comparative threshold cycle (2^−ΔΔCT^) method (*n* = 5 per group).

### Protein extraction and enzyme-linked immunosorbent assay

The nasal mucosa (8 mg) was resected and homogenized in 500 μL of CelLyticTM MT Cell Lysis Reagent (Sigma-Aldrich, Missouri, USA) with 5 μL of protease inhibitor cocktail (Sigma-Aldrich, Missouri, USA) and 120 U/mL of benzonase endonuclease (Sigma-Aldrich, Missouri, USA). The homogenates were centrifuged at 10,000×*g* at *T*_A_ for 5 min, following which the supernatants were obtained and stored frozen at − 80 °C before analysis. Blood samples (60 μL/mouse) were collected from the tail vein at day 3, day 9, or day 21, and allowed to clot at *T*_A_ for 30 min. Serum supernatant was obtained after centrifugation at 10,000×*g* for 5 min at *T*_A_.

The protein levels of IL-1β, IL-6, TNF-α, and IFN-γ in the samples were measured using the Mouse ELISA Kit (Invitrogen, Massachusetts, USA) according to the manufacturer’s instructions. The absorbance at 450 nm was measured using a microplate reader (VersaMax, Molecular Devices, California, USA).

### Statistical analysis

Statistical analyses were conducted using SPSS statistical software (SPSS Inc, Illinois, USA). The Mann–Whitney *U* test was used to assess differences between the Control and Poly(I:C) groups as well as for comparison between the right and left sides. Data were plotted as the means ± standard error of the mean. Significance was defined as a *P* value of less than 0.05.

## Results

### Poly(I:C) treatment induces loss of olfactory sensory neurons

**Fig. 2:**
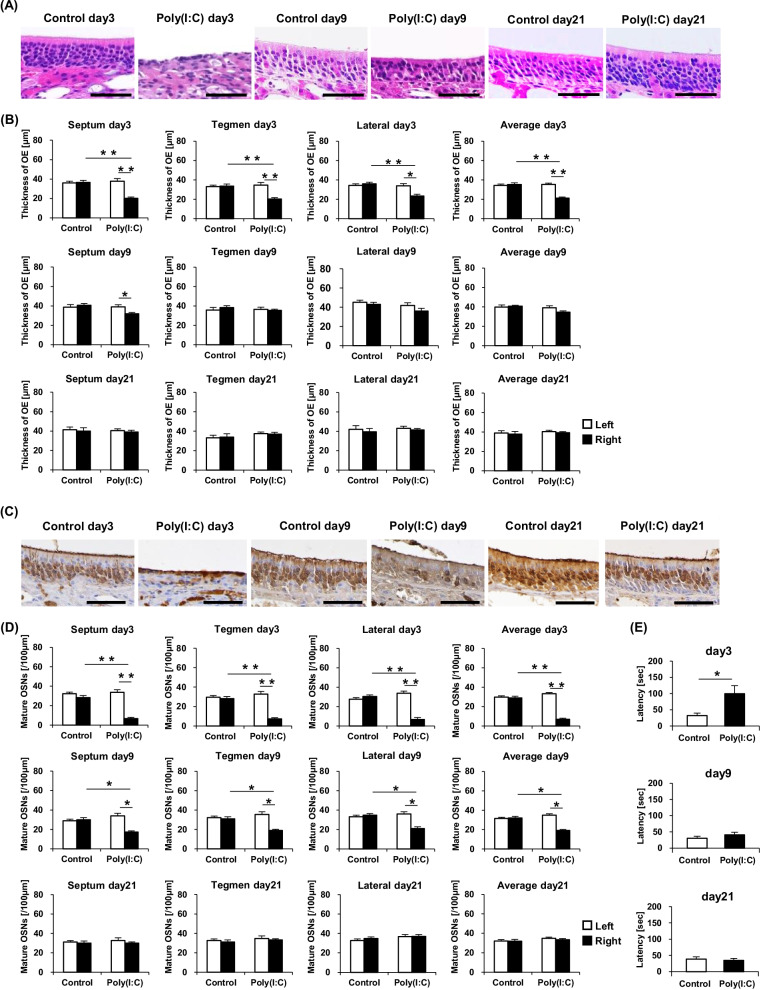
**A** Hematoxylin and eosin-stained sections, showing the OE thickness in the right nasal septum of the Control day 3, Poly(I:C) day 3, Control day 9, Poly(I:C) day 9, Control day 21, and Poly(I:C) day 21 groups (Scale bar = 50 μm). **B** Comparison of the OE thicknesses in the nasal septum, tegmen, and lateral area, respectively, at day 3, day 9, and day 21 among the groups. **C** Immunostained sections, showing mature OSNs in the right nasal septum of the Control day 3, Poly(I:C) day 3, Control day 9, Poly(I:C) day 9, Control day 21, and Poly(I:C) day 21 groups (Scale bar = 50 μm). **D** Comparison of the number of mature OSNs in the three-described areas at day 3, day 9, and day 21 among the studied groups. **E** Comparison of the latency [sec] to find the buried food at day 3, day 9, and day 21 between the Control and Poly(I:C) groups. Statistical analysis was performed using the Mann–Whitney *U* test, with *P* < 0.05 (*) and *P* < 0.01 (**) representing statistical significance. The error bars represent the standard error of the mean (*n* = 5). *OE* olfactory epithelium, *OSNs* olfactory sensory neurons, *Poly(I:C)* polyinosinic–polycytidylic acid

The impact of TLR3 stimulation with poly(I:C) on the OSNs was analyzed histologically. Upon examination of the right sides at day 3, the thicknesses of the OEs lining the nasal septum, tegmen, and lateral area, respectively, and the average OE thickness were observed to be significantly more reduced in the Poly(I:C) group than in the Control group. At day 9, there were no significant differences in the thicknesses of the right-side OEs between the groups. Left–right comparison within the Poly(I:C) group at day 3 revealed the thicknesses of the OEs lining the three described areas as well as the average OE thickness to be significantly decreased on the right side compared with the left side. By contrast, in the Poly(I:C) group at day 9, only the OE of the nasal septum was significantly thinner on the right side than on the left. At day 21, no significant differences in the thicknesses of the OEs were observed in any comparison. Representative images of the OE in the nasal septum area at day 3, day 9, and day 21 are shown in Fig. 2A, and the results are summarized in Fig. 2B.

Observation of the right sides at days 3 and 9 revealed that the number of OMP-positive cells, the density of mature OSNs in the OEs lining the nasal septum, tegmen, and lateral area, respectively, and the average OMP-positive cell count had declined significantly in the Poly(I:C) group compared with the Control group. In addition, left–right comparison within the Poly(I:C) group demonstrated the right side to have a significantly lower number of OMP-positive cells in the OEs of the three described areas and on average than the left side both at day 3 and day 9. At day 21, no significant differences in the number of OMP-positive cells were observed in any comparison. Representative images of the nasal septum at day 3, day 9, and day 21 are shown in Fig. 2C, and the results are summarized in Fig. 2D.

For evaluation of olfactory perceptual function, buried food tests were performed. At day 3, latency to find the buried food was significantly longer in the Poly(I:C) group than in the Control group. At day 9 and day 21, there were no significant differences in the latency between the groups. These results are shown in Fig. 2E.

### T cells and neutrophils are elevated in the olfactory mucosa of poly(I:C)-treated mice

**Fig. 3:**
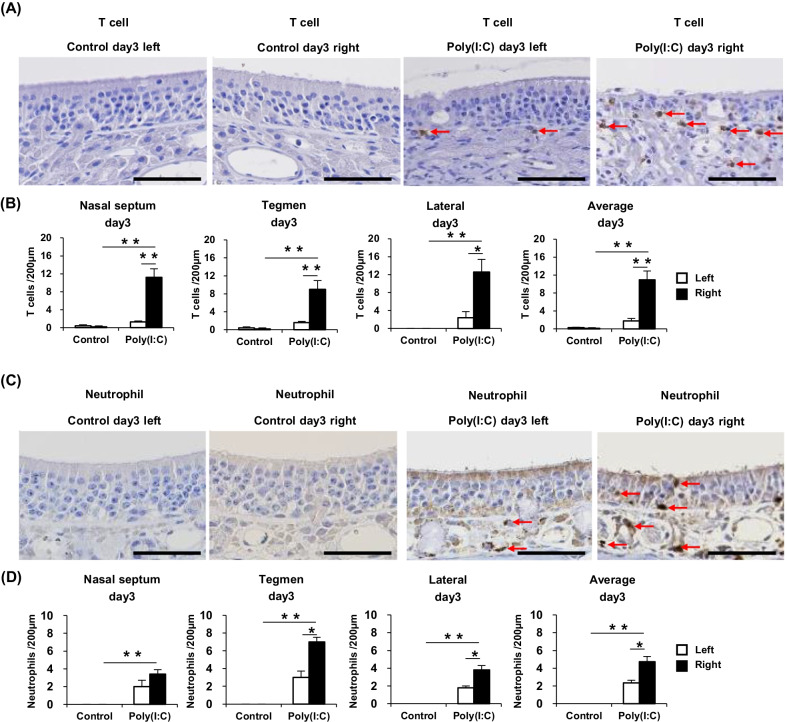
**A** Immunostained sections showing T-cell infiltration into the nasal septum of the Control day 3 and Poly(I:C) day 3 groups (Scale bar = 50 μm). **B** Comparison of the T-cell counts in the nasal septum, tegmen, and lateral areas between the groups. **C** Immunostained sections showing neutrophil infiltration into the tegmen of the Control day 3 and Poly(I:C) day 3 groups (Scale bar = 50 μm). **D** Comparison of the neutrophil counts in the three described areas between the groups. Statistical analysis was performed using the Mann–Whitney *U* test, with *P* < 0.05 (*) and *P* < 0.01 (**) representing statistical significance. The error bars represent the standard error of the mean (*n* = 5). Poly(I:C), polyinosinic–polycytidylic acid

Immunostaining with the anti-CD3 antibody was carried out to identify infiltrated T cells in the olfactory nasal mucosa. The numbers of T cells in the nasal septum, tegmen, and lateral area of the right side were individually calculated, totally averaged, and closely compared between the two mouse groups. Within each group, left–right differences in T-cell counts were also compared. Representative images of the nasal septum are shown in Fig. 3A. At day 3, the number of T cells in each area and the average T-cell count of the all three areas were significantly higher in the Poly(I:C) group than in the Control group at day 3. Moreover, at day 3, the individual T-cell counts of the three areas and their overall average were significantly higher in the right side than in the left side in the Poly(I:C) group. These results are summarized in Fig. 3B. At day 9 and day 21, there were very few T cells in the olfactory mucosa even in the Poly(I:C) group (data not shown).

Similarly, immunostaining with the anti-Ly-6G/Ly-6C antibody was carried out to detect infiltrated neutrophils, which were then counted and compared between the different groups. Representative images of the tegmen are shown in Fig. 3C. Compared with the Control group, the Poly(I:C) group displayed significantly greater individual and overall average numbers of neutrophils in the three areas at day 3. Moreover, in the Poly(I:C) group at day 3, the neutrophil counts of the tegmen and lateral area and the overall average count were significantly higher in the right side than in the left side. These results are summarized in Fig. 3D. At day 9 and day 21, there were very few neutrophils in the olfactory mucosa even in the Poly(I:C) group (data not shown).

### Microglia/macrophages are increased in the olfactory bulb of poly(I:C)-treated mice

**Fig. 4:**
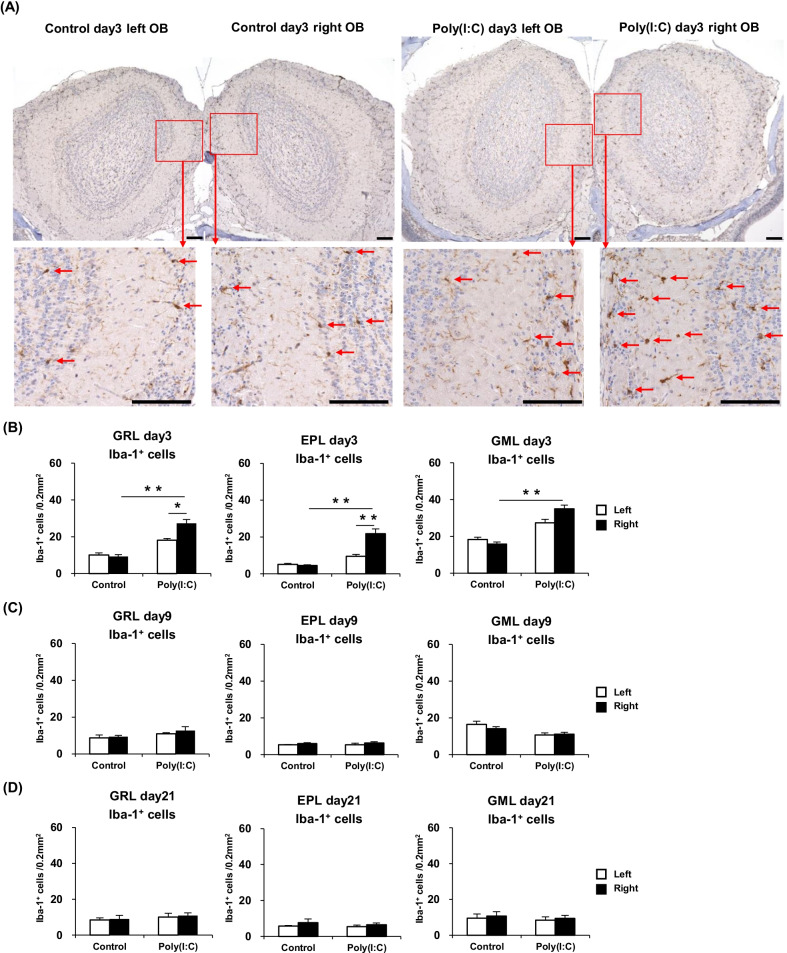
**A** Immunostained sections, showing the microglia/macrophages in the OB of the Control day 3 and Poly(I:C) day 3 groups (Scale bar = 100 μm). **B** Comparison of the Iba-1^+^ cell counts at day 3 in the GML, GRL, and EPL between the groups. **C** Comparison of the Iba-1^+^ cell counts at day 9 in the three described areas between the groups. **D** Comparison of the Iba-1^+^ cell counts at day 21 in the three described areas between the groups. Statistical analysis was performed using the Mann–Whitney *U* test, with *P* < 0.05 (*) and *P* < 0.01 (**) representing statistical significance. The error bars shown represent the standard error of the mean (*n* = 5). *EPL* external plexiform layer, *GML* glomerular layer, *GRL* granule layer, *OB* olfactory bulb, *OSNs* olfactory sensory neurons; *Poly(I:C)* polyinosinic–polycytidylic acid

Immunostaining with the anti-Iba-1 antibody was carried out to identify microglia/macrophages in the OB [20]. The numbers of Iba-1^+^ cells (microglia/macrophages) in the GRL, EPL, and GML of the right and left sides of the OB were individually calculated and closely compared between the mouse groups. Representative images of the OB Iba-1^+^ cells at day 3 are shown in Fig. 4A. The Iba-1^+^ cell count at day 3 in each area of the right side of the OB was significantly higher in the Poly(I:C) group than in the Control group. Left–right comparison within the Poly(I:C) group at day 3 showed that Iba-1^+^ cell counts in the GRL and EPL were significantly higher in the right side than in the left side. These results are summarized in Fig. 4B. At day 9 and day 21, the number of the Iba-1^+^ cells in each area of the OB was not significantly different between the Poly(I:C) and Control group. These results are summarized in Fig. 4C, D.

**Fig. 5:**
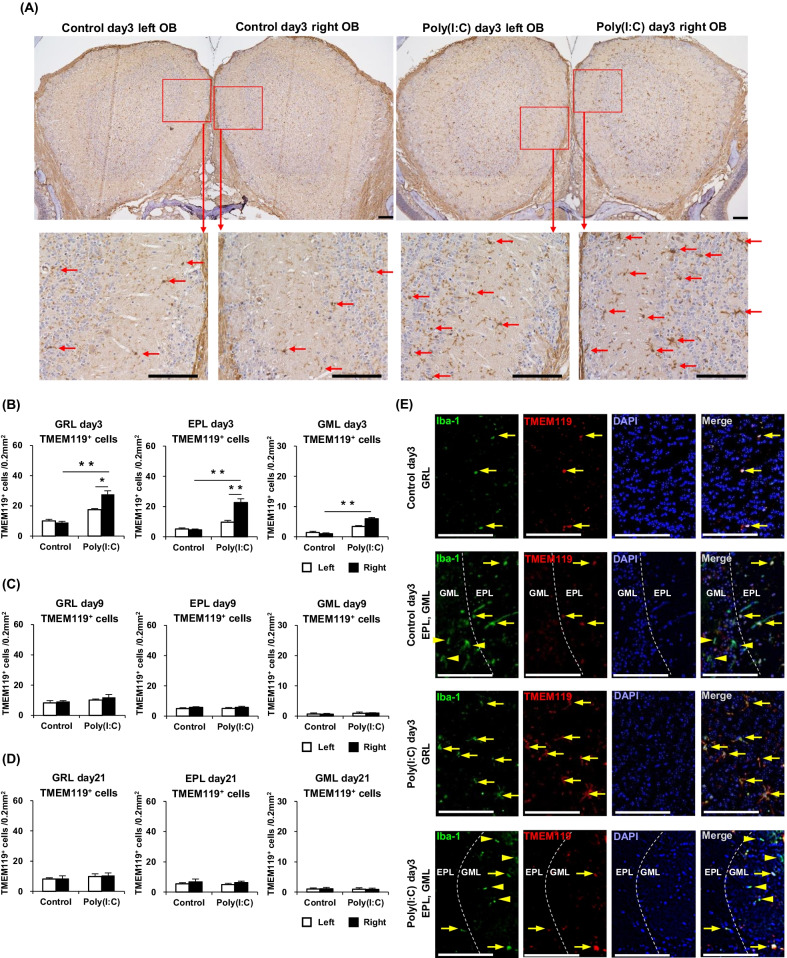
**A** Immunostained sections, showing TMEM119^+^ cells in the OB of the Control day 3 and Poly(I:C) day 3 groups (Scale bar = 100 μm). **B** Comparison of the TMEM119^+^ cell counts at day 3 in the GML, GRL, and EPL between the groups. **C** Comparison of the TMEM119^+^ cell counts at day 9 in the three described areas between the groups. **D** Comparison of the TMEM119^+^ cell counts at day 21 in the three described areas between the groups. **E** Immunostained sections, showing TMEM119^+^/Iba-1^+^ cells (arrow) and TMEM119^−^/Iba-1^+^ cells (arrowhead) in the GRL, EPL, and GML of the Control day 3 and Poly(I:C) day 3 groups (Scale bar = 100 μm). Statistical analysis was performed using the Mann–Whitney *U* test, with *P* < 0.05 (*) and *P* < 0.01 (**) representing statistical significance. The error bars shown represent the standard error of the mean (*n* = 5). *EPL* external plexiform layer, *GML* glomerular layer, *GRL* granule layer, *OB* olfactory bulb, *OSNs* olfactory sensory neurons, *Poly(I:C)* polyinosinic–polycytidylic acid

Next, immunostaining with the anti-TMEM119 antibody was employed to identify only microglia in the OB [20]. The numbers of TMEM119^+^ cells (microglia) in GRL, EPL, and GML of the right and left sides of the OB were individually calculated and closely compared between the mouse groups. Representative images of the OB TMEM119^+^ cells at day 3 are shown in Fig. 5A. The TMEM119^+^ cells at day 3 in each area of the right side of the OB were significantly increased in the Poly(I:C) group than in the Control group. Left–right comparison within the Poly(I:C) group at day 3 showed that TMEM119^+^ cell counts in the GRL and EPL were significantly higher in the right side than in the left side. At day 9 and day 21, the TMEM119^+^ cell count in each area of the OB was not significantly different between the Poly(I:C) and Control group. In the GRL and EPL, the numbers of TMEM119^+^ cells were almost the same as those of Iba-1^+^ cells. In the GML of the Poly(I:C) group at day 3, some TMEM119^+^ cells were confirmed, albeit the numbers of them were considerably less than those of Iba-1^+^ cells. In the Poly(I:C) group after day 9 and the Control group, very few TMEM119^+^ cells were observed in the GML. These results are summarized in Fig. 5B–D.

In addition, the ratio of TMEM119^+^ cells to Iba-1^+^ cells in the OB was qualitatively evaluated by immunofluorescence double staining with anti-Iba-1 antibody and anti-TMEM119 antibody. In the GRL and EPL, TMEM119 was expressed on almost all the Iba-1^+^ cells, suggesting that Iba-1^+^ cells in the GRL and EPL were microglia. On the other hand, most of Iba-1^+^ cells in the GML were TMEM119-cells, that is, macrophages. Representative images of immunofluorescence double staining at day 3 are shown in Fig. 5E.

### Astrocytes in the olfactory bulb are slightly activated by poly(I:C) intranasal treatment

**Fig. 6:**
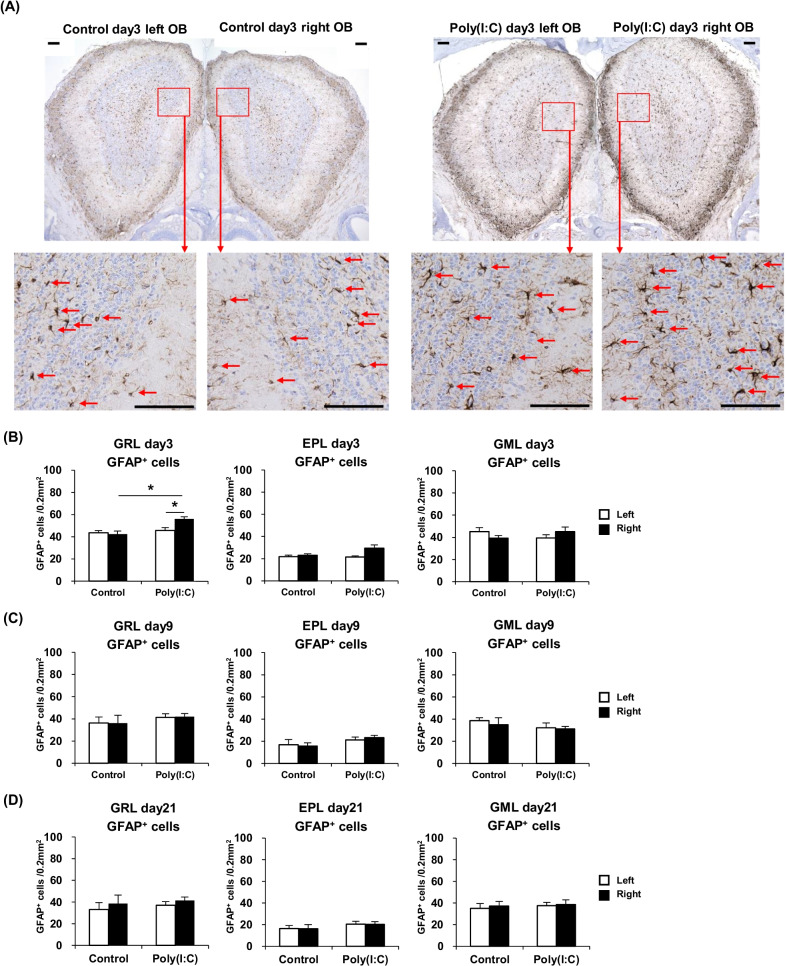
**A** Immunostained sections, showing activated astrocytes in the OB of the Control day 3 and Poly(I:C) day 3 groups (Scale bar = 100 μm). **B** Comparison of the GFAP^+^ cell counts at day 3 in the GML, GRL, and EPL between the groups. **C** Comparison of the GFAP^+^ cell counts at day 9 in the three described areas between the groups. **D** Comparison of the GFAP^+^ cell counts at day 21 in the three described areas between the groups. Statistical analysis was performed using the Mann–Whitney *U* test, with *P* < 0.05 (*) and *P* < 0.01 (**) representing statistical significance. The error bars represent the standard error of the mean (*n* = 5). *EPL* external plexiform layer, *GML* glomerular layer, *GRL* granule layer, *OB* olfactory bulb, *OSNs* olfactory sensory neurons, *Poly(I:C)* polyinosinic–polycytidylic acid

Immunostaining with the anti-GFAP antibody was carried out to detect activated astrocytes in the OB [21], which were then counted and compared between the different mouse groups. Representative images of the OB astrocytes are shown in Fig. 6A. At day 3, the number of GFAP-positive activated astrocytes in the right side of the OB was significantly higher in the GRL only in the Poly(I:C) group relative to that in the Control group. Left–right comparison within the Poly(I:C) group at day 3 showed that the astrocyte count was significantly larger in the GRL only of the right side than of the left side. These results are summarized in Fig. 6B. At day 9 and day 21, there were no significant differences in the GFAP-positive cell count of each area between the two mouse groups. These results are summarized in Fig. 6C, D.

### Proinflammatory cytokines are elevated not only in the nasal mucosa but also in the olfactory bulb

**Fig. 7:**
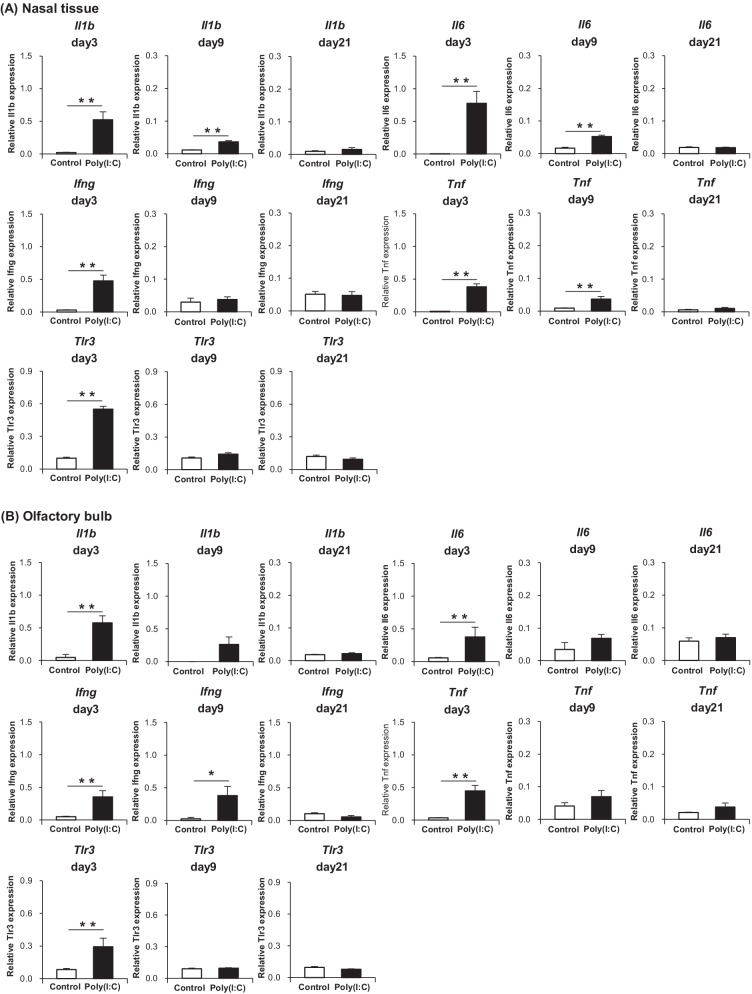
**A** Gene expression levels of proinflammatory cytokines and TLR3 in the nasal tissue. **B** Gene expression levels of proinflammatory cytokines and TLR3 in the olfactory bulb. Statistical analysis was performed using the Mann–Whitney *U* test, with *P* < 0.05 (*) and *P* < 0.01 (**) representing statistical significance. The error bars shown represent the standard error of the mean (*n* = 5). *Poly(I:C)* polyinosinic–polycytidylic acid

The gene expression levels of proinflammatory cytokines in the nasal tissue and OB were analyzed by qPCR. The results of the nasal tissue and OB are shown in Fig. 7A, B, respectively. At day 3, the expression levels of *Il1b*, *Il6*, *Tnf*, and *Ifng* in both the nasal tissue and OB were significantly elevated in the Poly(I:C) group compared with the levels in the Control group. At day 9, the expression levels of *Il1b*, *Il6*, and *Tnf* in the nasal mucosa were significantly higher in the Poly(I:C) group than in the Control group, albeit less remarkable than that at day 3. In the OB at day 9, only the expression level of *Ifng* was significantly higher in the Poly(I:C) group, where its degree of elevation was as much as that at day 3. At day 21, no significant difference was observed in the expression level of proinflammatory cytokines between the groups. The gene expression of *Tlr3* in the nasal tissue and OB was also confirmed by qPCR. The results of the nasal tissue and OB are shown in Fig. 7A, B, respectively. At day 3, the expression level of *Tlr3* both in the nasal tissue and OB turned out to be significantly higher in the Poly(I:C) group than in the Control group, suggesting that TLR3-expressing immune cells, such as macrophages [10] and microglia [22] are increased in the tissue. At day 9 and day 21, no significant difference was observed between the groups.

**Fig. 8:**
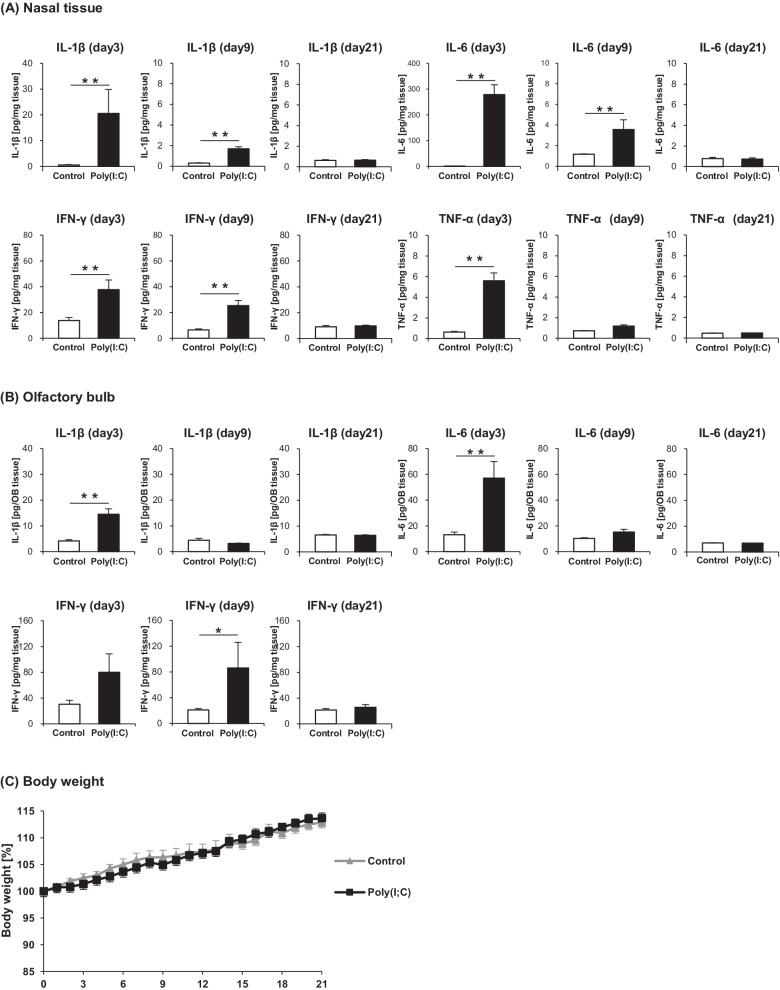
**A** Protein expression levels of proinflammatory cytokines in the nasal tissue. **B** Protein expression levels of proinflammatory cytokines in the olfactory bulb. **C** Body weight changes from day 0. Statistical analysis was performed using the Mann–Whitney *U* test, with *P* < 0.05 (*) and *P* < 0.01 (**) representing statistical significance. The error bars represent the standard error of the mean (*n* = 5). *Poly(I:C)* polyinosinic–polycytidylic acid

The protein levels of the proinflammatory cytokines in the nasal tissue and OB were quantified by ELISA. The results of the nasal tissue and OB are shown in Fig. 8A, B, respectively. The levels of IL-1β, IL-6, TNF-α, and IFN-γ in the nasal tissue were significantly higher in the Poly(I:C) group than in the Control group at day 3, whereas only the levels of IL-1β, IL-6, and IFN-γ were significantly higher in the Poly(I:C) group at day 9. In the OB, the IL-1β and IL-6 levels were significantly higher in the Poly(I:C) group than in the Control group at day 3 only. By contrast, IFN-γ level in the OB was higher in the Poly(I:C) group at both days 3 and 9, albeit a significant difference was observed only at day 9. TNF-α in the OB was not detected by ELISA. At day 21, there was no significant difference in the protein levels of cytokines between the groups. To assess the systemic effect of intranasal administration of poly(I:C), the levels of proinflammatory cytokines in the serum at day 3, day 9, and day 21 were also quantified by ELISA. However, none of IL-1β, IL-6, TNF-α, and IFN-γ in the serum was detected both in the Poly(I:C) group and the Control group. Furthermore, no significant difference was observed in the weight change rate from day 0 between the groups. The result of body weight monitoring is shown in Fig. 8C.

## Discussion

In the present study, we revealed that the microglial cells in the OB were increased, whereas the mature OSNs were decreased in a murine model of TLR3-mediated upper respiratory tract inflammation. We also demonstrated that both the gene and protein expression levels of the proinflammatory cytokines IL-1β, IL-6, TNF-α, and IFN-γ were elevated not only in the nasal mucosa but also in the OB of mice treated with the TLR3 ligand poly(I:C). In addition, we observed that only IFN-γ was continuously increasing in the OB, even after the histological changes in the olfactory mucosa and the levels of the other cytokines had almost recovered to control level at day 9. To the best of our knowledge, this is the first report to show increases in microglia and proinflammatory cytokines in the OB of mice with TLR3-mediated upper respiratory tract inflammation.

The olfactory system is unique in that peripheral OSNs are directly connected to the OB, the primary center of the system. The axons extending from the neuronal cell body in the nasal cavity pass through the cribriform plate and terminate in the OB, where they converge to form glomeruli and synaptic contacts with neurons resident in the OB [23]. Glial cells, such as microglia and astrocytes, are significant components of the OB, sustaining neuronal homeostasis [24]. These cells produce various kinds of effector molecules including cytokines, which have impact on neurogenesis and neuronal disorders [25]. Previous studies have suggested that proinflammatory cytokines, such as IL-1β and TNF-α, exert harmful effects on cell survival and decrease neurite outgrowth [26]. These cytokines also lead to microglial activation and reduce hippocampal neurogenesis [26].

The microglial cells are the immune cells of the central nervous system (CNS), where they play roles in recognition and scavenging of dead cells and pathogens [27]. They are also involved in various neural activities. Under normal physiological conditions, the microglial cells remain in the resting phenotype involved in neuronal activities, such as synaptogenesis, neurogenesis, and the release of neurotrophic factors [28]. However, when the neuronal system is injured and the homeostasis of the microenvironment is disturbed, the microglial cells shift into an active phenotype secreting cytokines [29]. There are two phenotypes of activated microglia: M1 and M2 [30, 31]. Under the action of IFN-γ, the resting microglia adopt the M1 phenotype via the classical activation pathway [32] and release the proinflammatory cytokines IL-1ß, IL-6, TNF-α, and IFN-γ [33, 34], which generally have negative effects on neurogenesis by inhibiting the proliferation or survival of new cells [35–38]. By contrast, transformation into the M2 microglia is induced by the cytokines IL-4 and IL-13 through the alternative activation pathway [39]. Contrary to their M1 counterparts, the M2 microglial cells operate mainly in neuroprotection and the reconstruction of neural networks in the CNS [30, 40].

In the present study, mice treated intranasally with poly(I:C) showed a higher density of microglia/macrophages in the OB and an increase in proinflammatory cytokines not only in the nasal mucosa but also in the OB as compared with the levels in the control mice. There are only a few reports describing the impact of intranasal stimulus-induced nasal inflammation on the OB. Hasegawa-Ishii et al. [41] showed that the microglia/macrophages were increased in the OB of mice treated intranasally with lipopolysaccharide (LPS). They also observed atrophy of the OB of the mice with LPS-stimulated chronic inflammation [42]. Herbert et al. [43] reported an increase in the OB microglia/macrophages in response to intranasal bacterial challenge. However, there are no reports to date that have investigated the link between TLR3 stimulation in the nasal mucosa and the immunological state of the OB. It is well known that TLR3 stimulation triggers the release of proinflammatory cytokines from several cell types, including nasal epithelial cells [44–47]. Our results are consistent with this current knowledge. In addition, it has been pointed out that peripheral afferent neurons can convey inflammatory factors, such as cytokines, to the projection neurons of the CNS, which may further transmit the inflammation to deeper regions [48–50]. These findings suggest that the increased cytokines in the olfactory mucosa may be transmitted to the OB along the olfactory nerve or trigeminal nerve that connect peripheral OSNs to the OB [51], inducing microglial activation and proliferation. Another possible mechanism of microglial activation is via the phagocytosis of degenerative neurons, as indicated in a report showing such activation by the phagocytosis of neuron debris [52]. In the present study, the significant damage to the OSNs is thought to lead to an increase in degenerative neuron debris, thereby resulting in the activation of the OB microglia.

It was first revealed that the ratio of microglia and macrophages depended on the layer of the OB. Almost all Iba-1^+^ cells in the GRL and the EPL were TMEM119^+^ microglial cells, while most of Iba-1^+^ cells in the GML were TMEM119-cells, that is, macrophages. There is no report discussing the ratio of microglia and macrophages in the OB. Martin et al. [20] showed that microglia were much more abundant than macrophages in the brain by flow cytometry. Our results are consistent with this report. Because the GML is the closest to peripheral olfactory nerve among the layers of the OB, macrophages may be much more abundant than microglia in the GML.

It is interesting that only IFN-γ in the OB was continuously increased even after the histological changes of the OE and OB microglia had almost returned to their original states at day 9. IFN-γ is generally considered a proinflammatory cytokine released from activated microglia and involved in the pathology of neuroinflammation [53, 54]. However, there are reports indicating that IFN-γ induces neuronal differentiation [55, 56]. In our study, IFN-γ was detected to be at a higher level than the other cytokines in the OB of the control mice. This cytokine may fluctuate slowly, because it has an important role even in the steady state.

The histological changes in the olfactory mucosa and the elevation of the proinflammatory cytokines in the nasal tissue were much more remarkable at day 3 than at day 9. In another study that addressed the histological changes of the olfactory epithelium in a rodent model of viral respiratory infection, the most severely damaged OSNs were observed 3 days after infection, and recovered considerably at day 10 [57]. From these results, we can posit that the course of poly(I:C)-induced upper airway inflammation in our study may be relatively similar to that of a true viral respiratory tract infection.

In the poly(I:C)-treated mice, the activated astrocytes were slightly increased only in the GRL of the OB. Previous studies have indicated that some microglia-derived factors can trigger astrocyte activation and induce neuroinflammation [24, 58]. Kobayashi et al. [21] observed an increase in OB astrocytes in mice whose olfactory pathway had been mechanically differentiated. However, it can be inferred that the OB astrocytes are not as highly affected by TLR3 stimulation as the microglia are in the OB.

This study had one notable limitation. Although we focused on the TLR3-mediated immune response, real viral respiratory tract infection can induce various responses that could also contribute to the status of the olfactory system. Nonetheless, it is important to investigate each element of the immune responses, and other signaling pathways can be evaluated using the same methodology as used in this study.

## Conclusions

We demonstrated that the microglia and proinflammatory cytokines were increased in the OB of mice with TLR3-mediated upper respiratory tract inflammation. Among the elevated proinflammatory cytokines, only the level of IFN-γ increased continuously, even after the OB microglia had almost returned to the normal state. Our findings suggest that immunological changes in the OB may be involved in the pathogenesis of olfactory disorders following URIs.

## Data Availability

The data sets generated during and/or analysed during the current study are available from the corresponding author on reasonable request.

## Abbreviations

T_A_: Ambient temperature
CNS: Central nervous system
CD3: Cluster of differentiation 3
ELISA: Enzyme-linked immunosorbent assay
EPL: External plexiform layer
GFAP: Glial fibrillary acidic protein
GML: Glomerular layer
Gapdh: Glyceraldehyde 3-phosphate dehydrogenase
GRL: Granule layer
IFN-γ: Interferon gamma
Il-1β: Interleukin-1 beta
IL-6: Interleukin-6
Iba-1: Ionized calcium-binding adaptor molecule 1
Ly-6G/Ly-6C: Lymphocyte antigen 6 complex locus protein G6D/lymphocyte antigen 6 complex locus protein G6C
OB: Olfactory bulb
OE: Olfactory epithelium
OMP: Olfactory marker protein
OSNs: Olfactory sensory neurons
PBS: Phosphate-buffered saline
poly(I:C): Polyinosinic–polycytidylic acid
PVOD: Postviral olfactory dysfunction
qPCR: Quantitative real-time polymerase chain reaction
TLR3: Toll-like receptor 3
TMEM119: Tumor necrosis factor
TNF: Trans-membrane protein 119
URI: Upper respiratory tract infection
